# Omecamtiv Mecarbil in Systolic Heart Failure: Clinical Efficacy and Future Directions of a Novel Myosin-Activating Inotropic Agent

**DOI:** 10.7759/cureus.82128

**Published:** 2025-04-12

**Authors:** Mahmoud M Ramadan, Abdullah L Alshawi, Yasmeen A Mostafa, Mohammad T Al-Obeid, Mohammed Elmahal

**Affiliations:** 1 Cardiology, Faculty of Medicine, Mansoura University, Mansoura, EGY; 2 Clinical Sciences, College of Medicine, University of Sharjah, Sharjah, ARE; 3 General Practice, Public Health Centre, Dubai Health Authority, Dubai, ARE; 4 Diabetes and Endocrinology, Khartoum North Teaching Hospital, Khartoum, SDN

**Keywords:** atomic-hf, cardiac contractility, cosmic-hf, galactic-hf, heart failure, inotropic therapy, meteoric-hf, myosin activator, omecamtiv mecarbil, systolic dysfunction

## Abstract

Heart failure with reduced ejection fraction (HFrEF) remains a global health challenge, associated with high morbidity, mortality, and rising healthcare costs. Despite advances in guideline-directed therapy, many patients, especially those with severely reduced ejection fraction, remain symptomatic. Traditional inotropes, including β-agonists and phosphodiesterase inhibitors, are limited in chronic HFrEF due to risks of arrhythmias, calcium overload, and increased myocardial oxygen demand.

Omecamtiv mecarbil (OM) is a novel cardiac myosin activator that enhances systolic contraction by increasing the efficiency of actin-myosin interactions. It prolongs systolic ejection time and improves stroke volume without elevating intracellular calcium or energy consumption. Although theoretical concerns exist regarding impaired diastolic filling, clinical trials have not confirmed such adverse effects in most patients.

This narrative review discusses OM’s pharmacologic profile, clinical trial data, and its potential as an adjunct therapy in advanced HFrEF. While not yet guideline-recommended, OM may benefit patients who remain symptomatic despite optimal treatment.

## Introduction and background

The rising prevalence of heart failure (HF) underscores the urgent need for therapies that target its fundamental mechanisms. Currently, HF affects millions worldwide and imposes a significant burden on healthcare systems [1]. Addressing this unmet need involves identifying new therapeutic targets and optimizing existing ones. Omecamtiv mecarbil (OM), a selective cardiac myosin activator, is one such innovation developed specifically for heart failure with reduced ejection fraction (HFrEF). Despite advancements in neurohormonal modulation, such as β-blockers, mineralocorticoid receptor antagonists (MRAs), angiotensin receptor-neprilysin inhibitors (ARNIs), and sodium-glucose co-transporter 2 (SGLT2) inhibitors, many patients remain symptomatic, and no standard therapy directly improves myocardial contractility.

OM’s mechanism of action involves selective cardiac myosin activation [2]. OM binds selectively to the catalytic S1 domain of myosin, enhancing adenosine triphosphate (ATP) efficiency and force generation without simply accelerating hydrolysis [3]. It stabilizes the actin-bound, pre-power stroke conformation, increasing force-generating cross-bridges during systole without affecting the full cross-bridge cycle [4]. This small-molecule modulation of motor protein function improves contractility at the filament level without altering calcium signaling [5].

Conventional inotropes, like dobutamine and milrinone, enhance cardiac output by increasing calcium or cyclic adenosine monophosphate (cAMP) levels [6], but are linked to arrhythmias, ischemia, and long-term myocardial injury [7]. In contrast, OM increases systolic force by prolonging actin-myosin binding and improving the duty cycle without disrupting calcium homeostasis [8]. While OM shows a favorable safety profile, mild, asymptomatic troponin elevations observed in trials warrant further studies [9].

This narrative review was conducted to summarize and critically appraise existing preclinical and clinical evidence on OM in HFrEF. Literature was identified through a structured search of PubMed, Scopus, and Google Scholar databases using keywords such as “Omecamtiv Mecarbil,” “myosin activator,” “systolic heart failure,” and names of relevant trials (Acute Treatment With Omecamtiv Mecarbil to Increase Contractility in Acute Heart Failure (ATOMIC-HF), Chronic Oral Study of Myosin Activation to Increase Contractility in Heart Failure (COSMIC-HF), Global Approach to Lowering Adverse Cardiac Outcomes Through Improving Contractility in Heart Failure (GALACTIC-HF), Multicenter Exercise Tolerance Evaluation of Omecamtiv Mecarbil Related to Increased Contractility in Heart Failure (METEORIC-HF)). Additional references were obtained through manual citation tracking. No formal Preferred Reporting Items for Systematic reviews and Meta-Analyses (PRISMA) methodology was followed due to the narrative nature of this review. Emphasis was placed on including peer-reviewed articles, clinical trials, and relevant mechanistic studies published in English up to March 2025.

## Review

Background and rationale

Among the most fundamental challenges in contemporary cardiology is the search for pharmacologic agents that can safely and effectively increase myocardial contractile force in the setting of systolic HF. HF is a large and growing global health problem, affecting an estimated 64 million people worldwide [10]. A long-standing hypothesis links early impairments in cardiac contractility to the progressive nature of HF, suggesting that improving contractile performance might alter disease trajectory [11]. Understanding how drugs, or even lifestyle interventions, affect myocardial contractility may help expand therapeutic options. However, many historical attempts to increase cardiac output (COP) through inotropic therapy have failed to improve long-term outcomes, often due to adverse effects such as arrhythmias, calcium overload, and increased myocardial oxygen demand. Most existing inotropes act indirectly by modulating neurohormonal or calcium-handling pathways, with limited precision at the level of the contractile apparatus.

One example is the experimental overexpression of SERCA-2a, which has been shown to restore contractility at the cellular level in models of systolic HF. However, clinical translation has proven difficult due to delivery challenges and inconsistent results in human trials [12], highlighting the broader limitations of targeting calcium cycling. In the majority of patients, symptomatic HF represents the final common pathway of a wide range of myocardial pathologies that share impaired systolic contractility as a central feature, typically manifesting as hypokinesis or reduced ejection fraction (EF), rather than a uniform calcium-handling deficit.

Anterograde inotropic strategies aimed at enhancing contractility have long been pursued, particularly to counteract the decline in cardiac performance that marks the transition from compensated to decompensated HF. This transition is often accompanied by increased end-diastolic pressure and worsening hemodynamic status. Historically, treatment began with digitalis and other toxic plant-derived inotropes, followed by agents targeting sympathetic stimulation and invasive support therapies at advanced stages [5]. While these approaches offered symptomatic relief, they have generally failed to modify disease progression and are best viewed as palliative responses to pump failure.

Research on OM has extended beyond the myocardium to include vascular studies, such as preclinical investigations in rat mesenteric small arteries, which revealed that OM suppresses the displacement component of the myosin-working stroke [13]. While these findings originate from vascular smooth muscle, they offer mechanistic insights into the modulation of myosin-actin interaction kinetics. Although OM was developed specifically for cardiac myosin, these vascular studies help elucidate how small-molecule myosin activators may alter force generation by modifying cross-bridge dynamics, an effect potentially translatable to failing cardiomyocytes.

Interestingly, these results appear to challenge the conventional assumption that actomyosin detachment and displacement are strictly coupled to the power stroke of the myosin head. Instead, they suggest a more nuanced mechanism of actomyosin interaction, with implications for both muscle contraction and pharmacological modulation [14]. However, it remains established that, under saturating ATP concentrations, actomyosin detachment proceeds faster than the unloaded displacement associated with the force-generating stroke.

Although complex, these observations bear clinical significance. In failing hearts, reduced cross-bridge cycling efficiency contributes to impaired force generation. Experimental data indicate that OM’s effect on myosin kinetics, particularly its ability to prolong the strongly bound, force-producing state, may overcome this inefficiency. For example, after a force clamp, the delayed ATP hydrolysis and slower phosphate release observed in cardiomyocytes support a model in which increased cross-bridge attachment enhances thin filament activation. This cooperative activation results in greater force output per ATP molecule, a highly advantageous feature in the energy-constrained environment of HF [15].

In this context, OM does not simply accelerate ATP turnover but improves contractile force by stabilizing force-generating cross-bridge states and promoting cooperative activation of the sarcomere. These properties distinguish OM from conventional inotropes and help explain its potential to improve COP without increasing intracellular calcium or myocardial oxygen consumption.

From this perspective, it is notable that, until recently, there were no widely adopted inotropic therapies that act directly on the molecular motor of contraction-cardiac myosin, even though acto-myosin cross-bridge formation is the core of force generation in striated muscle. This gap highlights a major therapeutic opportunity. A drug capable of restoring contractile force directly at the level of myosin could, by improving systolic function without compromising cellular energetics or calcium homeostasis, plausibly improve both hemodynamics and quality of life (QOL) in patients with advanced HFrEF. Building on these considerations, the rationale for developing agents capable of producing a dose-dependent increase in myocardial contractility through direct action on the contractile apparatus rather than via neurohormonal or receptor-mediated pathways is now well established. The first clinically studied molecule to fulfill this approach is OM [13].

Pathophysiology of systolic heart failure

HF is a growing public health concern associated with high morbidity and mortality, which correlates directly with increasing hospitalization rates. By 2030, over eight million Americans aged 18 or older are projected to have HF, equivalent to approximately one in 33 individuals. Moreover, HF remains the leading cause of hospitalization among individuals aged 65 and above. The total economic burden of HF in the United States alone is expected to exceed $70 billion by 2030, encompassing both direct healthcare expenditures and indirect societal costs [16]. Despite this rising burden, the development of new therapeutic agents for HF has lagged significantly behind advances seen in other cardiovascular conditions.

Patients with HF experience progressive structural and functional deterioration of the myocardium, ultimately leading to decompensation, poor QOL, and a five-year survival rate of less than 50% following diagnosis [17]. While current pharmacological therapies, such as β-blockers, angiotensin-converting enzyme (ACE) inhibitors, ARNIs, MRAs, and SGLT2 inhibitors, offer substantial benefits by targeting neurohormonal pathways, they do not directly reverse the primary myocardial dysfunction. Instead, they largely modulate compensatory mechanisms such as sympathetic activation, renin-angiotensin system upregulation, and fluid retention, which temporarily preserve COP but often drive long-term maladaptive remodeling [18]. As a result, there remains a critical need for novel therapies that directly target the underlying deficit in myocardial contractile performance, the hallmark of systolic HF [19].

Systolic HF, defined by reduced EF <40% (HFrEF), is characterized by impaired left ventricular contraction, which lies at the core of its pathophysiology [20]. Although HF arises from diverse etiologies, including ischemic injury, pressure overload, and genetic cardiomyopathies, these conditions converge on a shared endpoint: insufficient myocardial contractility [21]. As contractility declines, COP determined by heart rate (HR) and stroke volume (SV) becomes inadequate to meet systemic metabolic demands. This insufficiency leads to hallmark HF symptoms such as fatigue, dyspnea, and exercise intolerance [22].

To compensate, the body initiates neurohormonal activation aimed at maintaining perfusion pressure and COP. Initially beneficial, these mechanisms, such as increased sympathetic tone and renin-angiotensin system activity, ultimately promote structural changes in the myocardium. This remodeling, intended to recapitulate aspects of fetal cardiac growth, leads to chamber dilation, myocardial hypertrophy, and reduced compliance [23]. Over time, these adaptations become maladaptive, exacerbating ventricular dysfunction and accelerating disease progression.

The limited ability of existing therapies to directly address myocardial contractile dysfunction, combined with their potential for adverse effects (e.g., hypotension, renal dysfunction, hyperkalemia), has driven interest in re-examining the fundamental pathophysiology of HF to identify new therapeutic targets [24]. Genetic predispositions, comorbidities (such as diabetes or hypertension), and lifestyle factors can all accelerate disease evolution and complicate treatment [25]. Despite compensatory changes, the failing heart often cannot meet the body’s demands, and its structural adaptations, initially meant to preserve function, ultimately become pathological. Thus, targeting the decline in contractile force at the sarcomeric level represents a promising approach to restoring mechanical function and improving clinical outcomes in HFrEF [23].

Inotropic mechanisms in heart failure

A central therapeutic dilemma in HF management is that traditional positive inotropic agents, such as catecholamines or phosphodiesterase inhibitors, are associated with increased mortality in chronic settings [26]. While these agents enhance myocardial contractility and temporarily improve COP, they do so at the expense of increased myocardial oxygen consumption, intracellular calcium overload, and heightened arrhythmic risk. In some animal models and patients with chronic systolic HF, sustained use of these drugs has been shown to accelerate disease progression and worsen long-term outcomes [27]. This risk is especially concerning in younger or ambulatory patients with HF who experience a high symptomatic burden but have limited therapeutic options.

Currently, cardiac resynchronization therapy (CRT) is the only non-pharmacological intervention proven to improve both contractility and survival in HF patients with reduced EF. However, its benefit is confined to individuals with specific electrical conduction abnormalities, such as left bundle branch block and QRS duration ≥130 ms [28]. Pharmacologic therapy has advanced substantially, and guideline-directed medical therapy now includes β-blockers, MRAs, ARNIs, and SGLT2 inhibitors such as dapagliflozin and empagliflozin. These therapies reduce both hospitalizations and mortality across the HFrEF spectrum. Nonetheless, a subset of patients remains symptomatic due to medication intolerance (e.g., hypotension, renal dysfunction), nonadherence, or refractory HF despite optimal therapy.

Newer agents such as levosimendan and OM have emerged as alternative inotropic strategies with distinct mechanisms. Levosimendan functions as a calcium sensitizer by stabilizing the calcium-bound conformation of troponin C and opening ATP-sensitive potassium (K-ATP) channels, contributing to vasodilation and potential cardioprotection [29]. In contrast, OM directly binds to cardiac myosin and increases the number of myosin heads in a force-generating state, thereby improving systolic function without increasing intracellular calcium or myocardial oxygen demand.

Phase II clinical trials demonstrated OM’s favorable hemodynamic and safety profile in both acute and chronic HF settings [30]. The large phase III GALACTIC-HF trial, which enrolled over 8,000 patients with HFrEF, found that OM modestly reduced the composite endpoint of cardiovascular death or HF events, particularly in patients with severely reduced EF (≤28%). However, no statistically significant reduction in all-cause mortality was observed, underscoring OM’s role as an adjunctive or symptom-targeted therapy rather than a definitive mortality-reducing agent. OM has not yet received broad regulatory approval and is not currently included in major HF guidelines.

In parallel, non-pharmacological strategies remain essential components of HF management. Guideline-based recommendations include daily sodium restriction (<2-3 g/day), fluid restriction in selected patients with hyponatremia or fluid overload, and structured aerobic exercise training to improve functional capacity. Advanced monitoring tools such as the CardioMEMS™ system (Abbott, IL, USA) used for remote pulmonary artery pressure monitoring have demonstrated reductions in HF hospitalizations, though their adoption in real-world practice is constrained by cost, infrastructure, and patient selection.

Emerging device-based therapies such as interatrial shunt devices and implantable coronary sinus reducers are currently under clinical investigation. These differ from CRT or mechanical circulatory support (e.g., left ventricular assist devices) in both mechanism and indications. Their long-term safety and efficacy remain to be fully determined through ongoing trials. Table 1 shows a comparison between OM and some common inotropic agents.

**Table 1:** Comparison of inotropic agents

Agent	Mechanism	Advantages	Disadvantages
β-agonists	Increase intracellular calcium	Rapid improvement in contractility	Increased arrhythmic risk, energy demand
Phosphodiesterase inhibitors	Increase cyclic adenosine monophosphate (cAMP) levels, leading to increased calcium	Improved contractility and vasodilation	Increased arrhythmic risk, hypotension
Omecamtiv mecarbil	Directly enhances myosin-actin interaction	No increase in calcium or energy demand	Limited mortality benefit, mild troponin elevations

Current therapeutic approaches

HF is a devastating clinical syndrome affecting an estimated 26 million people worldwide. It carries a poor prognosis, with a five-year mortality rate approaching 50% despite medical advances [31,32]. HF remains a progressive disease with high rates of hospital admissions and readmissions, both of which contribute to a significant decline in QOL and functional capacity [33]. This article reviews recent therapeutic advances in HF and highlights new pharmacologic and device-based strategies under investigation, including the selective cardiac myosin activator OM, which was recently evaluated in the phase III GALACTIC-HF trial [34].

Standard HF management includes lifestyle and pharmacologic interventions. According to current guideline-directed recommendations, patients are advised to follow a sodium-restricted diet (<2-3 g/day), restrict fluid intake in cases of hyponatremia or significant fluid overload, and participate in regular aerobic exercise (e.g., 20-30 minutes, three to five times per week) as tolerated. Smoking cessation and avoidance of excessive alcohol intake are also recommended [35].

Pharmacologic therapy includes β-blockers, ACE inhibitors or angiotensin II receptor blockers (ARBs), ARNIs (e.g., sacubitril/valsartan), MRAs, diuretics, and SGLT2 inhibitors such as dapagliflozin and empagliflozin, which are now cornerstone therapies for HFrEF due to their proven benefits in reducing HF hospitalization and cardiovascular mortality. Additional agents include ivabradine, which lowers HR and improves outcomes in selected patients with sinus rhythm and elevated resting HR despite β-blocker therapy.

Despite these treatments, not all patients achieve adequate control, which may be due to refractory HF (progressive symptoms despite optimal therapy), intolerance to medications (e.g., hypotension, renal dysfunction, electrolyte imbalances), or issues with adherence. In such cases, newer agents and technologies are being explored.

OM has shown promise by enhancing cardiac contractility through selective activation of cardiac myosin without increasing intracellular calcium or myocardial oxygen consumption. In the GALACTIC-HF trial, which enrolled over 8,000 patients with HFrEF, OM modestly reduced the composite endpoint of cardiovascular death or HF events, particularly in those with severe systolic dysfunction (EF ≤28%). However, it did not confer a significant reduction in all-cause mortality. OM is not yet included in major HF guidelines, and further research is underway to clarify its regulatory pathway and potential combination strategies.

Levosimendan, another investigational agent, acts via calcium sensitization and activation of K-ATP channels, offering both inotropic support and vasodilation [29]. Vericiguat, a soluble guanylate cyclase stimulator, was evaluated in Vericiguat in Patients with Heart Failure and Reduced Ejection Fraction (VICTORIA), a pivotal phase III trial evaluating vericiguat in patients with HFrEF and recent decompensation. It demonstrated a modest but statistically significant reduction in the composite outcome of cardiovascular death or HF hospitalization [36]. Notably, vericiguat was evaluated in patients with HF with preserved ejection fraction (HFpEF) (LVEF ≥45%) by the Vericiguat to Improve Physical Functioning in Daily Living Activities of Patients With Heart Failure and Preserved Ejection Fraction (VITALITY-HFpEF) trial, where no improvement in physical limitation using the Kansas City Cardiomyopathy Questionnaire (KCCQ) score or six-minute walk distance was found [37]. Evolocumab, a PCSK9 inhibitor, is not a direct HF treatment but plays a supportive role by improving lipid profiles and reducing atherosclerotic cardiovascular risk in HF patients with underlying ischemic heart disease. Vardenafil (a PDE5 inhibitor) has been evaluated for potential benefits in HFpEF, but evidence remains limited and largely mechanistic; further trials are needed to justify its use.

Device-based interventions are also evolving. The CardioMEMS™ system enables remote pulmonary artery pressure monitoring and has demonstrated reductions in HF-related hospitalizations. However, its real-world application is limited by cost, access disparities, and strict patient selection criteria. Investigational options such as interatrial shunt devices and implantable coronary sinus reducers are being evaluated for use in HFpEF and HFrEF, respectively. These differ from traditional CRT or left ventricular assist devices in both design and indication, and their long-term efficacy and safety are still under investigation in clinical trials [37-40].

Molecular mechanisms of omecamtiv mecarbil

The primary goal of drug therapy for systolic HF is to improve cardiac contractility. Traditional inotropes, such as β-agonists, phosphodiesterase inhibitors (inodilators), and calcium sensitizers, enhance the interaction between regulatory (troponin-tropomyosin) and contractile (actin-myosin) proteins by lowering the threshold for calcium-mediated activation [41,42]. However, this mechanism is often suboptimal due to its lack of muscle-type specificity. Activation of striated muscle in a non-selective fashion can inadvertently stimulate vascular and smooth muscle, potentially leading to undesirable hemodynamic effects [43].

OM represents a novel class of inotropic agents that bypass this limitation by acting directly on cardiac myosin. OM binds selectively to the β-myosin heavy chain isoform expressed in cardiac tissue, with minimal off-target activity in skeletal or smooth muscle [44]. This specificity allows OM to enhance contractile function without increasing intracellular calcium concentrations or myocardial oxygen consumption.

OM’s unique mechanism centers on prolonging the actomyosin attachment phase during the power stroke. By lowering the apparent dynamic spring constant of the myosin working stroke, OM effectively dampens and extends the duration of force generation, leading to longer cross-bridge binding [45]. This sustained interaction enables more efficient contractile force production per ATP molecule, thereby enhancing myocardial performance without altering calcium handling or inducing arrhythmias.

Additionally, OM promotes cooperative activation of the thin filament. It increases the number of strongly bound myosin heads, which reinforces adjacent troponin-tropomyosin units and facilitates further cross-bridge recruitment. Unlike conventional inotropes that rely on elevated intracellular calcium to activate the thin filament, OM leverages sarcomeric cooperativity, representing a fundamental shift in contractility modulation.

Preclinical studies

Insights from in vitro biochemical assays and in situ mechanical experiments have shed light on OM’s load-dependent behavior. Although OM slows adenosine diphosphate (ADP) release by approximately 20-fold, ATP still facilitates actin detachment under low-load conditions, preserving contractile responsiveness. However, at higher loads, OM-modified myosin resists the typical increase in detachment rate seen with elevated ATP, suggesting a stiffer and more load-resistant cross-bridge conformation [13]. This mechanical adaptation enables prolonged force maintenance during systole, aligning with the hemodynamic demands of failing hearts.

In animal models of HF, OM has been shown to increase myocardial contraction efficiency by stabilizing the force-generating state of myosin without altering myofibrillar phosphorylation or promoting calcium overload. These effects translate into improved ventricular performance with preserved energy efficiency [13,44].

Clinical implications

OM’s mechanism holds significant clinical potential for patients with HFrEF, particularly those who are intolerant to traditional inotropes or who experience side effects related to calcium overload and sympathetic stimulation. By enhancing SV and systolic function without increasing oxygen consumption or intracellular calcium, OM addresses the core mechanical deficiency in systolic HF while minimizing metabolic and electrophysiological risk.

Furthermore, OM’s reliance on thin filament cooperativity rather than calcium-driven activation suggests a safer, more targeted approach to improving COP. As research continues to explore its use in clinical populations, OM may become a key adjunctive therapy for patients with advanced HF, offering a mechanism distinct from current guideline-directed treatments.

Myosin activation and cardiac function

Maximal cardiac function depends on the optimal regulation of myocardial contractility. With each heartbeat, individual cardiac myocytes contract as myosin motor proteins bind to actin, hydrolyze ATP, and undergo the power stroke that generates mechanical force [46]. The total contractile output of the heart results from the spatial and temporal coordination of mechanical interactions across billions of sarcomeres, the fundamental contractile units of muscle, and the efficient transfer of mechanical force through the titin-myosin-actin system [13].

OM is a selective activator of cardiac myosin that increases the number of myosin heads in the pre-power stroke, strongly actin-bound state. This leads to more cross-bridges in force-generating conformations, ultimately increasing systolic force generation [47]. The force produced by myosin is transferred via the thick filament to the thin filament, and OM-induced binding promotes cooperative thin filament activation. This cooperative mechanism enhances overall contractile force output, contributing to increased COP in patients with systolic HF [47,48].

Historically, drug development efforts to improve contractility in systolic HF have focused on modulating cytosolic calcium to boost actin-myosin interaction. However, this approach has proven suboptimal due to significant drawbacks such as arrhythmias, increased myocardial oxygen consumption, and calcium overload-induced apoptosis, which can exacerbate ventricular remodeling and disease progression [49].

In contrast, OM targets sarcomeric function directly without altering intracellular calcium levels. Nevertheless, its unique mechanism is not entirely without potential side effects. Prolongation of the systolic ejection time and increased cross-bridge binding can, in some patients, lead to myocardial stiffness and may contribute to diastolic dysfunction, particularly if dosing is excessive or in patients with borderline diastolic reserve. These effects have been observed in both preclinical and clinical studies and underscore the importance of dosing precision and patient selection [50].

While enhanced myocardial force generation may be beneficial in the acute setting, such as the transitional period following hospitalization, its long-term impact remains complex. All else being equal, increased contractile force improves hemodynamics initially; however, persistent force augmentation without adequate relaxation may have a neutral or even adverse effect over time, especially in patients with comorbid diastolic dysfunction or elevated afterload [21].

Key clinical studies

Cleland and colleagues [51] conducted a double-blind, placebo-controlled, crossover, dose-ranging phase II trial to evaluate OM in 45 patients with stable systolic HF. The study population had a baseline left ventricular ejection fraction (LVEF) of ≤40%, reflecting moderate-to-severe systolic dysfunction. Patients received intravenous OM infusions for two, 24, or 72 hours with monitoring of vital signs, echocardiographic parameters, and plasma drug levels. The primary objective was safety and tolerability; secondary goals included assessment of dose response and determination of an optimal plasma concentration range. Results demonstrated concentration-dependent increases in systolic ejection time (up to 80 ms), SV (up to 9.7 mL), and EF, with accompanying reductions in LV volumes, findings suggestive of improved myocardial efficiency. A modest HR reduction (up to 2.7 bpm) was also observed. These effects were not uniformly dose-proportional, with more pronounced improvements in mid-to-higher dose groups. Blood pressure remained stable across most plasma levels, but mild systolic hypotension was reported at concentrations >500 ng/mL. At plasma levels exceeding 1300 ng/mL, two patients developed transient myocardial ischemia, likely due to excessive prolongation of systolic ejection time impairing diastolic coronary perfusion, a known limitation at supratherapeutic dosing. One patient overdosed to ~1750 ng/mL and experienced reversible ischemic symptoms. These findings underscored the need for tight therapeutic monitoring to maintain efficacy without compromising safety.

The Acute Treatment with Omecamtiv Mecarbil to Increase Contractility in Acute Heart Failure (ATOMIC-HF ) trial [52] was a randomized, placebo-controlled phase II study in 613 patients hospitalized for acute decompensated HFrEF (baseline LVEF ≤40%). The primary endpoint was dyspnea relief over 48 hours using a five-point Likert scale. OM failed to meet this primary outcome, highlighting the limitations of subjective endpoints in acute settings. However, secondary outcomes showed dose-dependent increases in systolic ejection duration and LVEF. Although no significant increase in arrhythmias or clinical HF deterioration was seen, higher doses were associated with increased serum troponin levels. These elevations likely reflected subclinical myocardial stress due to prolonged cross-bridge cycling but remained within the expected range for inotropic agents and did not correlate with adverse outcomes. Objective parameters such as N-terminal pro B-type natriuretic peptide (NT-proBNP) and pulmonary capillary wedge pressure were also evaluated, providing complementary hemodynamic insights.

The COSMIC-HF trial [53] was a phase II, double-blind, randomized study in 448 chronic HFrEF patients (LVEF ≤40%, New York Heart Association (NYHA) class II-III), assessing OM over 20 weeks. Patients received either OM (25 mg or 50 mg BID) or placebo. OM significantly improved LVEF (~3.6%), reduced LV end-systolic and end-diastolic volumes, and prolonged systolic ejection duration, all without increasing myocardial oxygen demand. HR was reduced (~5 bpm), and OM remained effective even in patients prone to hypotension, an important consideration for those receiving vasodilators or with advanced HF. Mild troponin elevations were observed in some patients but were not associated with adverse structural or arrhythmic events. While the trial duration was relatively short, these favorable hemodynamic effects formed the basis for the longer-term GALACTIC-HF trial. COSMIC-HF also supported the view that OM is best suited for patients with more advanced systolic dysfunction, guiding patient selection in future studies.

The Global Approach to Lowering Adverse Cardiac Outcomes Through Improving Contractility in Heart Failure (GALACTIC-HF) trial [9,34,54] was a phase III, randomized, double-blind study of 8,256 patients with HFrEF (LVEF ≤35%). Patients received OM or placebo alongside standard therapy. The primary composite endpoint (time to first HF event or cardiovascular death) showed a statistically significant 8% reduction, primarily driven by a reduction in HF hospitalizations. Mortality was not significantly reduced, suggesting that improved contractility alone may not overcome factors like residual fibrosis, neurohormonal activation, or irreversible myocardial damage. Subgroup analyses showed the most benefit in patients with LVEF ≤28%, pointing toward OM’s potential role in advanced HF phenotypes. Compared to traditional inotropes like dobutamine or milrinone, which increase mortality via pro-arrhythmic and oxygen-demand mechanisms, OM enhances contractility without elevating intracellular calcium or triggering arrhythmias, representing a safer long-term inotropic strategy.

In comparison, SGLT2 inhibitors (e.g., dapagliflozin, empagliflozin) have demonstrated robust benefits in both HFrEF and HFpEF populations, including reductions in mortality and HF hospitalizations across a broad spectrum of patients regardless of diabetes status. Their mechanism, mediated through natriuresis, improved myocardial metabolism and reduced inflammation, complementing OM’s contractility-focused action and possibly offering synergistic potential when used together. Likewise, vericiguat, a soluble guanylate cyclase stimulator approved for HFrEF patients with recent decompensation, improves outcomes by enhancing nitric oxide signaling and reducing vascular resistance. Though its benefit on mortality is modest, vericiguat offers another strategy for stabilizing high-risk patients post-discharge. In contrast to OM, vericiguat does not directly affect myocardial contractility but rather improves hemodynamic status via vascular pathways [36,37]. These comparisons highlight OM’s niche as a direct sarcomere-targeted inotrope that may be best suited as an adjunctive therapy in patients with severely reduced LVEF, particularly when traditional neurohormonal therapies are optimized but symptoms persist or contractile reserve remains impaired.

The METEORIC-HF trial [55], a phase III randomized, placebo-controlled study, evaluated OM’s impact on peak oxygen consumption (VO₂ max) in patients with chronic HFrEF. Over 20 weeks, OM failed to improve exercise capacity, despite clear improvements in systolic function. This finding underscores the concept that enhancing cardiac contractility alone is insufficient to restore functional capacity in HF patients. Exercise intolerance in HF is multifactorial and often driven by skeletal muscle dysfunction, reduced mitochondrial efficiency, and impaired peripheral oxygen extraction, all of which may persist despite improved central hemodynamics.

The absence of VO₂ max improvement limits OM’s role in addressing this key clinical burden. As such, OM is best considered a hemodynamic enhancer that may benefit targeted subgroups, particularly those with severely reduced LVEF and low COP. However, to address exercise intolerance more comprehensively, OM may need to be combined with adjunctive therapies that target skeletal muscle perfusion, microvascular dysfunction, or mitochondrial bioenergetics, such as SGLT2 inhibitors, exercise training, or novel metabolic modulators. This combination approach may help bridge the gap between improved cardiac performance and actual improvements in patient-centered outcomes like exertional capacity and QOL.

The findings and limitations of the key clinical trials on OM are summarized in Table 2.

**Table 2:** Key clinical trials on omecamtiv mecarbil ATOMIC-HF: Acute Treatment With Omecamtiv Mecarbil to Increase Contractility in Acute Heart Failure; COSMIC-HF: Chronic Oral Study of Myosin Activation to Increase Contractility in Heart Failure; GALACTIC-HF: Global Approach to Lowering Adverse Cardiac Outcomes Through Improving Contractility in Heart Failure; METEORIC-HF: Multicenter Exercise Tolerance Evaluation of Omecamtiv Mecarbil Related to Increased Contractility in Heart Failure; HFrEF: heart failure with reduced ejection fraction; LVEF: left ventricular ejection fraction

Trial	Phase	Population	Key Findings	Limitations
ATOMIC-HF	II	Acute HFrEF	Improved systolic ejection duration; no significant dyspnea relief	Limited symptom improvement
COSMIC-HF	II	Chronic HFrEF	Increased LVEF, reduced LV volumes; no increase in arrhythmias	Mild troponin elevations
GALACTIC-HF	III	Chronic HFrEF (LVEF ≤35%)	Reduced HF hospitalizations; no significant mortality benefit	Limited mortality benefit
METEORIC-HF	III	Chronic HFrEF	No significant improvement in exercise capacity	Limited functional improvement

Safety profile and adverse effects

What are the general thoughts on OM and its potential as a future therapy for HF with reduced EF (LVEF ≤40%, or ‘systolic HF’)? The latter has emerged as a global health challenge, partly due to increasing life expectancy and aging populations. Although current therapies reduce morbidity and mortality, residual risk remains high, with mortality rates still ranging between 40-50%. Despite this burden, pharmaceutical investment in novel HFrEF therapies has been limited, and relatively few agents have produced positive trial outcomes [56].

OM is a selective cardiac myosin activator, not an inodilator. Unlike phosphodiesterase inhibitors or catecholamines, OM does not cause vasodilation, nor does it alter intracellular calcium handling. Instead, OM enhances contractility by increasing the duty ratio of myosin heads in the force-generating state. It binds to the catalytic domain of the myosin head (S1) and allosterically to the S2 segment (the elastic component of the myosin tail), thereby stabilizing the converter domain and promoting an open configuration of the actin-binding clefts [47,57].

Clinicians are increasingly required to evaluate OM’s safety profile and understand its adverse effects based on available clinical trial data. As OM enters broader clinical use, monitoring strategies become essential. The most commonly observed adverse effect is a mild, dose-related increase in serum troponin, which has not been associated with adverse clinical outcomes or structural myocardial damage [9]. Nonetheless, clinicians may consider checking troponin levels at baseline and periodically during dose titration, particularly in high-risk or symptomatic patients.

OM has been associated with dose-dependent reductions in HR and prolongation of systolic ejection time, which generally plateau at therapeutic plasma concentrations. However, plasma levels above 1300 ng/mL have been linked to transient myocardial ischemia, likely due to excessive prolongation of systole leading to impaired coronary perfusion. These findings underscore the importance of therapeutic drug monitoring to maintain plasma concentrations within a defined safety window.

Other laboratory abnormalities observed during clinical trials include mild increases in alanine aminotransferase (ALT), transient decreases in estimated glomerular filtration rate (eGFR), and elevations in white blood cell (WBC) count [54,58]. These changes were generally mild and self-limiting, with no clear association with clinical adverse events. While the mechanism underlying eGFR decline is not fully elucidated, it is presumed to reflect hemodynamic shifts, such as increased afterload or altered renal perfusion, rather than direct nephrotoxicity. Periodic monitoring of liver function (e.g., ALT) and renal function (e.g., serum creatinine/eGFR) may be prudent during initiation and dose adjustment, especially in patients with baseline hepatic or renal impairment.

Importantly, across major trials, OM has not been associated with increased incidence of arrhythmias, hypotension, or sustained myocardial ischemia, distinguishing it from traditional inotropes and supporting its favorable safety profile for long-term use in selected HFrEF patients. Table 3 summarizes the main adverse effects encountered with OM.

**Table 3:** Summary of omecamtiv mecarbil adverse effects ALT: alanine aminotransferase; eGFR: estimated glomerular filtration rate

Adverse Effect	Incidence	Clinical Implications
Troponin elevations	Mild	Possible subclinical myocardial injury
Reduced heart rate	Dose-dependent	Generally well-tolerated
Increased ALT	Rare	Monitor liver function
Decreased eGFR	Rare	Monitor renal function

Future directions and potential applications

Ongoing research continues to explore novel aspects of OM’s mechanism and its potential utility beyond the treatment of HFrEF, LVEF ≤40%. While preclinical and clinical trials have primarily focused on HFrEF, OM’s distinctive sarcomeric mechanism-enhancing contractility without increasing cytosolic calcium or stimulating adrenergic signaling positions it as a novel adjunctive option, particularly in select patient populations. Potential areas of future exploration include dilated cardiomyopathy, where sarcomeric dysfunction is central; constrictive pericarditis, where modest improvements in systolic output may offset diastolic restriction; and peri-transplant settings, such as in marginal donor hearts or post-operative graft dysfunction.

OM’s use in functional mitral regurgitation (MR) is mechanistically appealing, as it could enhance forward SV. However, by prolonging systolic ejection time, OM may paradoxically increase regurgitant volume, particularly in patients with significant MR and non-compliant left atria. As such, its role in MR remains speculative and warrants rigorous clinical investigation.

Interest also surrounds OM’s potential interaction with myosin isoform expression. In human myocardium, the β-myosin heavy chain (V3) is predominant, especially in failing hearts where α-myosin (V1) is downregulated. OM selectively targets β-myosin, which may explain its efficacy in HFrEF and suggests isoform-specific responsiveness in advanced systolic dysfunction [59]. Further studies are needed to determine whether myosin isoform profiling could help tailor therapy.

The GALACTIC-HF trial, with a median follow-up of 21.8 months, already provides meaningful insight into OM’s chronic use [34]. While OM achieved an 8% relative risk reduction in the composite of cardiovascular death or HF events, this was primarily driven by reductions in HF hospitalizations. Importantly, there was no significant mortality benefit, underscoring that OM should be viewed as a complementary therapy rather than a transformative breakthrough. Notably, subgroup analyses revealed the greatest benefit in patients with LVEF ≤28%, supporting the concept of OM as a targeted therapy for those with advanced systolic dysfunction and impaired contractile reserve.

Though OM avoids many of the limitations of traditional inotropes, such as calcium overload and pro-arrhythmic effects, its safety profile warrants careful interpretation. Clinical trials have reported mild, dose-dependent troponin elevations, modest increases in ALT, transient reductions in eGFR, and occasional elevations in white blood cell counts. These effects were generally not clinically significant, but their long-term implications, particularly regarding myocardial fibrosis, diastolic dysfunction, or subclinical inflammation, remain unclear. As such, routine monitoring of troponin, renal function (feGFR), and liver enzymes may be warranted in select patients during chronic therapy.

Future research should also prioritize evaluating OM in combination with established disease-modifying therapies, such as SGLT2 inhibitors, ARNIs, and beta-blockers. SGLT2 inhibitors offer metabolic and renal benefits, while ARNIs improve neurohormonal balance, mechanistically complementing OM’s direct contractile effect. However, co-administration with beta-blockers warrants caution, given the potential for additive bradycardia. Pharmacodynamic studies are needed to determine optimal sequencing, synergy, or contraindications in such multidrug regimens.

In this context, large-scale, real-world registries and post-marketing surveillance studies will be critical for refining OM’s role within guideline-directed medical therapy, optimizing patient selection, and defining its true long-term benefit-risk balance.

## Conclusions

OM offers a novel approach to treating systolic HF by directly enhancing cardiac myosin activity without increasing intracellular calcium or myocardial oxygen demand. Unlike traditional inotropes, OM improves systolic function through a calcium-independent mechanism, making it a potentially safer adjunctive option. Clinical trials, including ATOMIC-HF, COSMIC-HF, GALACTIC-HF, and METEORIC-HF, have shown OM's ability to improve left ventricular function and reduce HF hospitalizations. 

Future research should explore OM in combination with therapies like SGLT2 inhibitors and ARNIs while carefully evaluating interactions with beta-blockers. With further validation, OM may emerge as a valuable, targeted option for patients with severely reduced EF within a personalized HF treatment paradigm.

## References

[1] Savarese G, Becher PM, Lund LH, Seferovic P, Rosano GM, Coats AJ (2023). Global burden of heart failure: a comprehensive and updated review of epidemiology. Cardiovasc Res.

[2] Teerlink JR, Diaz R, Felker GM (2021). Cardiac myosin activation with omecamtiv mecarbil in systolic heart failure. N Engl J Med.

[3] Brunello E, Fusi L, Ghisleni A, Park-Holohan SJ, Ovejero JG, Narayanan T, Irving M (2020). Myosin filament-based regulation of the dynamics of contraction in heart muscle. Proc Natl Acad Sci U S A.

[4] Hashem S, Tiberti M, Fornili A (2017). Allosteric modulation of cardiac myosin dynamics by omecamtiv mecarbil. PLoS Comput Biol.

[5] Kaplinsky E, Mallarkey G (2018). Cardiac myosin activators for heart failure therapy: focus on omecamtiv mecarbil. Drugs Context.

[6] Francis GS, Bartos JA, Adatya S (2014). Inotropes. J Am Coll Cardiol.

[7] Felker GM, O'Connor CM (2001). Inotropic therapy for heart failure: an evidence-based approach. Am Heart J.

[8] Patel PH, Nguyen M, Rodriguez R, Surani S, Udeani G (2021). Omecamtiv mecarbil: a novel mechanistic and therapeutic approach to chronic heart failure management. Cureus.

[9] Teerlink JR, Diaz R, Felker GM (2020). Omecamtiv mecarbil in chronic heart failure with reduced ejection fraction: rationale and design of GALACTIC-HF. JACC Heart Fail.

[10] Shahim B, Kapelios CJ, Savarese G, Lund LH (2023). Global public health burden of heart failure: an updated review. Card Fail Rev.

[11] Ge Z, Li A, McNamara J, Dos Remedios C, Lal S (2019). Pathogenesis and pathophysiology of heart failure with reduced ejection fraction: translation to human studies. Heart Fail Rev.

[12] Zhihao L, Jingyu N, Lan L (2020). SERCA2a: a key protein in the Ca(2+) cycle of the heart failure. Heart Fail Rev.

[13] Woody MS, Greenberg MJ, Barua B, Winkelmann DA, Goldman YE, Ostap EM (2018). Positive cardiac inotrope omecamtiv mecarbil activates muscle despite suppressing the myosin working stroke. Nat Commun.

[14] Franz P, Ewert W, Preller M, Tsiavaliaris G (2020). Unraveling a force-generating allosteric pathway of actomyosin communication associated with ADP and PI release. Int J Mol Sci.

[15] Nabiev SR, Ovsyannikov DA, Tsaturyan AK, Bershitsky SY (2015). The lifetime of the actomyosin complex in vitro under load corresponding to stretch of contracting muscle. Eur Biophys J.

[16] Braunwald E (2013). Heart failure. JACC Heart Fail.

[17] G-CHF Investigators (2023). Global variations in heart failure etiology, management, and outcomes. JAMA.

[18] Severino P, D'Amato A, Prosperi S (2023). Heart failure pharmacological management: gaps and current perspectives. J Clin Med.

[19] Correale M, Tricarico L, Fortunato M, Mazzeo P, Nodari S, Di Biase M, Brunetti ND (2021). New targets in heart failure drug therapy. Front Cardiovasc Med.

[20] Murphy SP, Ibrahim NE, Januzzi JL Jr (2020). Heart failure with reduced ejection fraction: a review. JAMA.

[21] Ponikowski P, Voors AA, Anker SD (2016). 2016 ESC Guidelines for the diagnosis and treatment of acute and chronic heart failure: the Task Force for the diagnosis and treatment of acute and chronic heart failure of the European Society of Cardiology (ESC)Developed with the special contribution of the Heart Failure Association (HFA) of the ESC. Eur Heart J.

[22] Beard DA, Hummel SL, Jezek F (2022). Heart failure as a limitation of cardiac power output. Function (Oxf).

[23] Hartupee J, Mann DL (2017). Neurohormonal activation in heart failure with reduced ejection fraction. Nat Rev Cardiol.

[24] Patel C, Deoghare S (2015). Heart failure: novel therapeutic approaches. J Postgrad Med.

[25] Skrzynia C, Berg JS, Willis MS, Jensen BC (2015). Genetics and heart failure: a concise guide for the clinician. Curr Cardiol Rev.

[26] Maack C, Eschenhagen T, Hamdani N (2019). Treatments targeting inotropy. Eur Heart J.

[27] Tisdale JE, Patel R, Webb CR, Borzak S, Zarowitz BJ (1995). Electrophysiologic and proarrhythmic effects of intravenous inotropic agents. Prog Cardiovasc Dis.

[28] Thomas G, Kim J, Lerman BB (2019). Improving cardiac resynchronisation therapy. Arrhythm Electrophysiol Rev.

[29] Psotka MA, Teerlink JR (2017). Direct myosin activation by omecamtiv mecarbil for heart failure with reduced ejection fraction. Handb Exp Pharmacol.

[30] Lim GB (2017). Heart failure: Phase II trial results of omecamtiv mecarbil. Nat Rev Cardiol.

[31] Savarese G, Lund LH (2017). Global public health burden of heart failure. Card Fail Rev.

[32] Bytyçi I, Bajraktari G (2015). Mortality in heart failure patients. Anatol J Cardiol.

[33] Rodríguez-Artalejo F, Guallar-Castillón P, Pascual CR (2005). Health-related quality of life as a predictor of hospital readmission and death among patients with heart failure. Arch Intern Med.

[34] Felker GM, Solomon SD, Claggett B (2022). Assessment of omecamtiv mecarbil for the treatment of patients with severe heart failure: a post hoc analysis of data from the GALACTIC-HF randomized clinical trial. JAMA Cardiol.

[35] Dugal JK, Malhi AS, Ramazani N, Yee B, DiCaro MV, Lei K (2024). Non-pharmacological therapy in heart failure and management of heart failure in special populations—a review. J Clin Med.

[36] Armstrong PW, Pieske B, Anstrom KJ (2020). Vericiguat in patients with heart failure and reduced ejection fraction. N Engl J Med.

[37] Armstrong PW, Lam CS, Anstrom KJ (2020). Effect of vericiguat vs placebo on quality of life in patients with heart failure and preserved ejection fraction: the VITALITY-HFpEF randomized clinical trial. JAMA.

[38] Knowler WC, Fowler SE, Hamman RF (2009). 10-year follow-up of diabetes incidence and weight loss in the Diabetes Prevention Program Outcomes Study. Lancet.

[39] Sabatine MS, Giugliano RP, Keech AC (2017). Evolocumab and clinical outcomes in patients with cardiovascular disease. N Engl J Med.

[40] Redfield MM, Chen HH, Borlaug BA (2013). Effect of phosphodiesterase-5 inhibition on exercise capacity and clinical status in heart failure with preserved ejection fraction: a randomized clinical trial. JAMA.

[41] Rao YJ, Xi L (2009). Pivotal effects of phosphodiesterase inhibitors on myocyte contractility and viability in normal and ischemic hearts. Acta Pharmacol Sin.

[42] Bistola V, Arfaras-Melainis A, Polyzogopoulou E, Ikonomidis I, Parissis J (2019). Inotropes in acute heart failure: from guidelines to practical use: therapeutic options and clinical practice. Card Fail Rev.

[43] Overgaard CB, Dzavík V (2008). Inotropes and vasopressors: review of physiology and clinical use in cardiovascular disease. Circulation.

[44] Nagy L, Kovács Á, Bódi B (2015). The novel cardiac myosin activator omecamtiv mecarbil increases the calcium sensitivity of force production in isolated cardiomyocytes and skeletal muscle fibres of the rat. Br J Pharmacol.

[45] Rohde JA, Thomas DD, Muretta JM (2017). Heart failure drug changes the mechanoenzymology of the cardiac myosin powerstroke. Proc Natl Acad Sci U S A.

[46] Sugiura S (1999). Actin—myosin interaction. Cardiovasc Res.

[47] Planelles-Herrero VJ, Hartman JJ, Robert-Paganin J, Malik FI, Houdusse A (2017). Mechanistic and structural basis for activation of cardiac myosin force production by omecamtiv mecarbil. Nat Commun.

[48] Nakanishi T, Oyama K, Tanaka H (2022). Effects of omecamtiv mecarbil on the contractile properties of skinned porcine left atrial and ventricular muscles. Front Physiol.

[49] Kass DA, Solaro RJ (2006). Mechanisms and use of calcium-sensitizing agents in the failing heart. Circulation.

[50] Zhou S, Liu Y, Huang X, Wu C, Pórszász R (2024). Omecamtiv mecarbil in the treatment of heart failure: the past, the present, and the future. Front Cardiovasc Med.

[51] Cleland JGF, Teerlink JR, Senior R (2011). The effects of the cardiac myosin activator, omecamtiv mecarbil, on cardiac function in systolic heart failure: a double-blind, placebo-controlled, crossover, dose-ranging phase 2 trial. Lancet.

[52] Teerlink JR, Felker GM, McMurray JJ (2016). Acute Treatment With Omecamtiv Mecarbil to Increase Contractility in Acute Heart Failure: The ATOMIC-AHF Study. J Am Coll Cardiol.

[53] Teerlink JR, Felker GM, McMurray JJ (2016). Chronic Oral Study of Myosin Activation to Increase Contractility in Heart Failure (COSMIC-HF): a phase 2, pharmacokinetic, randomised, placebo-controlled trial. Lancet.

[54] Metra M, Pagnesi M, Claggett BL (2022). Effects of omecamtiv mecarbil in heart failure with reduced ejection fraction according to blood pressure: the GALACTIC-HF trial. Eur Heart J.

[55] Lewis GD, Voors AA, Cohen-Solal A (2022). Effect of Omecamtiv Mecarbil on Exercise Capacity in Chronic Heart Failure With Reduced Ejection Fraction: The METEORIC-HF Randomized Clinical Trial. JAMA.

[56] McCullough PA (2017). How trialists and pharmaceutical sponsors have failed us by thinking that acute heart failure is a 48-hour illness. Am J Cardiol.

[57] Swenson AM, Tang W, Blair CA (2017). Omecamtiv mecarbil enhances the duty ratio of human β-cardiac myosin resulting in increased calcium sensitivity and slowed force development in cardiac muscle. J Biol Chem.

[58] Alqatati F, Elbahnasawy M, Bugazia S (2022). Safety and efficacy of omecamtiv mecarbil for heart failure: a systematic review and meta-analysis. Indian Heart J.

[59] Cheng W, Cui C, Liu G (2023). NF-κB, a potential therapeutic target in cardiovascular diseases. Cardiovasc Drugs Ther.

